# *Plasmodium falciparum* Nicotinamidase as A Novel Antimalarial Target

**DOI:** 10.3390/biom12081109

**Published:** 2022-08-12

**Authors:** Dickson Donu, Chiranjeev Sharma, Yana Cen

**Affiliations:** 1 Department of Medicinal Chemistry, Virginia Commonwealth University, Richmond, VA 23219, USA; 2 Institute for Structural Biology, Drug Discovery and Development, Virginia Commonwealth University, Richmond, VA 23219, USA

**Keywords:** *P. falciparum*, nicotinamidase, antimalarial, NAD^+^ homeostasis

## Abstract

Inhibition of *Plasmodium falciparum* nicotinamidase could represent a potential antimalarial since parasites require nicotinic acid to successfully recycle nicotinamide to NAD^+^, and importantly, humans lack this biosynthetic enzyme. Recently, mechanism-based inhibitors of nicotinamidase have been discovered. The most potent compound inhibits both recombinant *P. falciparum* nicotinamidase and parasites replication in infected human red blood cells (RBCs). These studies provide evidence for the importance of nicotinamide salvage through nicotinamidase as a central master player of NAD^+^ homeostasis in *P. falciparum*.

## 1. Introduction

**Figure 1:**
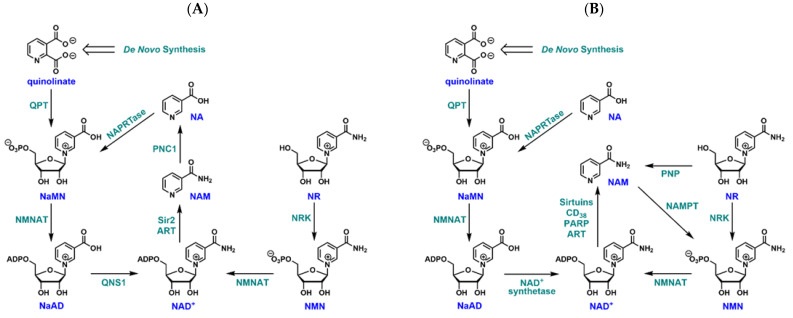
Comparison of NAD^+^ biosynthetic pathways. (**A**) NAD^+^ metabolism in most bacteria, yeast and multicellular eukaryotes. NA: nicotinic acid; NAM: nicotinamide; NR: nicotinamide riboside; NMN: nicotinamide mononucleotide; NaMN: nicotinic acid mononucleotide; NaAD: nicotinic acid adenine dinucleotide; QPT: quinolinate phosphoribosyltransferase; PNC1: nicotinamidase; QNS1: glutamine-dependent NAD^+^ synthetase; NRK: nicotinamide riboside kinase; ART: ADP-ribosyl transferase; NMNAT: nicotinamide mononucelotide adenylyltransferase. (**B**) NAD^+^ biosynthetic pathways in mammals. There is a lack of nicotinamidase, no direct path for converting nicotinamide to nicotinic acid. NAMPT: nicotinamide phosphoribosyltransferase; PNP: purine nucleoside phosphorylase; PARP: poly(ADP-ribose) polymerase.

Nicotinamidases are metabolic enzymes that catalyze the hydrolysis of nicotinamide (NAM) to nicotinic acid (NA) [1,2]. NAM is the reactive moiety within the dinucleotides NAD(H) and NADP(H). The conversion of free NAM to NA catalyzed by nicotinamidase enables cells to recycle this moiety, leading to the re-conjugation of pyridine-3-carboxylate and ribose-5-phosphate to form nicotinic acid mononucleotide (NaMN), and eventual reformation of NAD^+^ (Figure 1). Nicotinamidases are widely distributed in biology, and are found ubiquitously in archaea, mycobacteria, eubacteria, and in unicellular and multicellular eukaryotes, but not in mammals [1,3,4,5,6,7,8,9,10]. The fact that nicotinamidases are not found in human cells, but appear in numerous pathogens that infect humans, suggests that nicotinamidases could be the targets of novel antimicrobials.

**Figure 2:**
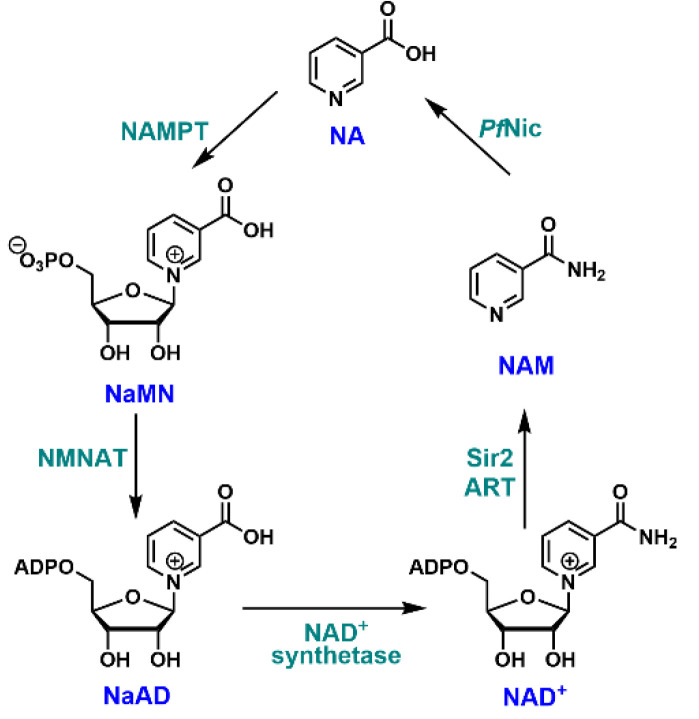
NAD^+^ biosynthetic pathways in *P. falciparum* as identified by activity, transcriptome, and genome studies on the organism.

To better understand the feasibility of targeting nicotinamidase as an antimicrobial strategy in general, and how it might be of particular effect in treating *P. falciparum* infection, it is instructive to consider the general organization of a complete NAD^+^ metabolism in microbes (Figure 1) and how it contrasts with humans (Figure 2). The typical pathways that converge to NAD^+^ are divided into several components. These are widely designated de novo and salvage pathways [3,4,7,11]. All known de novo pathways require the formation of quinolinate (Figure 1 and Figure 2). Quinolinate is the precursor of the biosynthetic intermediate NaMN which implies that NA is not formed as a free compound in the de novo pathway. NaMN is then adenylated to nicotinic acid adenine dinucleotide (NaAD) and further acted upon by ATP-dependent NAD^+^ synthetase to form NAD^+^. Both microbes and humans have this pathway. Because of the overlapping of these pathways, the identification of highly selective inhibitors that do not affect human enzymes has been a major concern, even though enzymes such as nicotinate mononucleotide adenylyltransferase [12,13] and NAD^+^ synthetase [14,15] have been identified as potential targets for antimicrobials.

Salvage pathways are known to reconstitute NAD^+^ from metabolites that already incorporate a finished NAM or NA ring within the compound. Within most organisms, the NAM and NA pathways are overlapping, with the enzyme nicotinamidase required to convert NAM to NA (Figure 1A) [3,7]. A nicotinic acid phosphoribosyltransferase (NAPRTase) is responsible for resynthesis of NaMN, with the remaining steps common to the *de novo* pathway. The corresponding strategy of human metabolism is different. NAM is directly recycled by nicotinamide phosphoribosyltransferase (NAMPT) that couples it to 5-phospho-ribose-1-pyrophosphate (PRPP) to form nicotinamide mononucleotide (NMN, Figure 1B) [11,16], a mechanism first recognized by Detrich et al. in 1966 [17]. NA is recycled to form NaMN, in what is known as the Preiss–Handler pathway, [18] reaching a common intermediate of the de novo pathway [8,19]. Importantly, human NAD^+^ metabolism is not known to create NA as a free species. It is thought that NA originates mostly from dietary sources and is not synthesized in cells [8,19]. Because NAM is formed readily from rapid NAD^+^ turnover in mammalian cells, the most prevalent precursor to make NAD^+^ in the body is NAM. Importantly, the only salvage enzyme that is unique to pathogens is nicotinamidase, suggesting that if microbes are heavily reliant on this enzyme for NAD^+^ biosynthesis, it could be an effective antimicrobial target.

The protozoan parasite *Plasmodium falciparum* causes the most severe form of human malaria and utilizes antigenic variation to avoid antibody recognition, resulting in greatly extended periods of infection [20,21,22]. Drug resistance is widespread [23,24,25], and novel agents against new targets are needed to support combination-therapy approaches promoted by the World Health Organization (WHO) [26,27]. The pathways of NAD^+^ metabolism in *P. falciparum* have not been extensively characterized, but genome and transcriptome analyses indicate that the NAD^+^ metabolism of this microbe is restricted to NA and NAM salvage pathways (Figure 2) [28,29,30]. Because *P. falciparum* has nicotinamidase and nicotinate phosphoribosyltransferase (NPT), it has been suggested that it can utilize either NA or NAM as the NAD^+^ source [30]. However, given that human cells do not directly make free NA and that NAM is abundant in mammalian tissues and in cells [31,32], *P. falciparum* has been Implied to be highly dependent upon nicotinamidase activity for NAD^+^ synthesis. Consistent with this notion, *P. falciparum* infected erythrocytes express high levels of nicotinamidase, and have NAD^+^ contents 10 times higher than uninfected cells [33]. In support of the hypothesis that nicotinamidase activity is required for the bulk of NAD^+^ synthesis in *P. falciparum*, we present the novel result that NAM is the preferred source of *P. falciparum* NAD^+^ biosynthesis, even in the presence of NA. The inhibition of *P. falciparum* nicotinamidase (*Pf*Nic) has a significant adverse effect on NAD^+^ levels and parasite growth in cultures. Overall, our study suggests that *Pf*Nic serves as a viable antimalarial drug target.

## 2. Materials and Methods

### 2.1. Reagents and Instruments

All reagents were purchased from Sigma-Aldrich (St. Louis, MO, USA) or Fisher Scientific (Pittsburgh, PA, USA) and were of the highest purity commercially available. HPLC was performed on a Dionex Ultimate 3000 HPLC system equipped with a diode array detector using Macherey-Nagel C18 reverse-phase column. Radiolabeled samples were counted in a Beckman LS6500 scintillation counter. NMR spectra were acquired on a Bruker AVANCE III 500 MHz high-field NMR spectrometer, and the data were processed using Topspin software (https://www.bruker.com/en/products-and-solutions/mr/nmr-software/topspin.html?gclid=EAIaIQobChMIi-T93OvA-QIVzmSLCh2ScAJOEAAYASAAEgLEgvD_BwE, accessed on 2 July 2022). Mass spectra were obtained with a Bruker Autoflex II MALDI-TOF mass spectrometer operated in positive ion reflectron mode. HRMS spectra were acquired with either a Waters Micromass Q-tof Ultima or a Thermo Scientific Q-Exactive hybrid Quadrupole Orbitrap.

### 2.2. Cloning and Expression of PfNic and Synthesis of Inhibitor

*P. falciparum* nicotinamidase was cloned, expressed, and purified as described previously [34]. 5-Me-nicotinaldehyde was synthesized according to previously published methods [34].

### 2.3. Parasite Culture

The chloroquine-sensitive *P. falciparum* strain 3D7 was cultivated at 5% hematocrit in RPMI 1640 medium, 0.5% Albumax II (Invitrogen, Waltham, MA, USA), 0.25% sodium bicarbonate, and 0.1 mg/mL gentamicin. Parasites were incubated at 37 °C in an atmosphere of 5% oxygen, 5% carbon dioxide and 90% nitrogen. Parasite cultures were synchronized using percoll/sorbitol gradient centrifugation as described previously [35]. Briefly, 3 mL of 40% percoll/6% sorbitol/RPMI 1640 medium was layered over 3 mL of 70% percoll/6% sorbitol/RPMI 1640 medium in a 15 mL centrifuge tube. Infected RBCs at 50% hematocrit were layered on top of the gradient. The gradient was then centrifuged at 12,000× *g* for 20 min at room temperature. Highly synchronized parasites were recovered from the 40%/70% interface, washed twice with complete culture media. Microscopic evaluation of the recovered cells verify that they were >95% infected mature-stage parasites (trophozoites and schizonts). These cells were reconstituted with fresh RBCs and media. Following erythrocyte invasion, the synchronized culture was used for drug or precursor treatment [36].

### 2.4. Inhibitor or NAD^+^ Precursor Treatment and NAD^+^ Measurements

Synchronized parasite cultures were treated with either inhibitor or NAD^+^ precursor at indicated concentrations. Media were changed every 12 h along with the chemicals. After a total of 48 h incubation, parasites were harvested using percoll/sorbitol gradient described above and washed twice with complete culture media. The pellets were then washed with 1 mL of PBS and were resuspended in 1 mL of PBS for cell counting using hemocytometer. After counting, the cells were centrifuged at 4000 rpm for 3 min at room temperature. To the pellets were added standard ^18^O-NAD^+^ (typically 1 nmol) and 70 μL of ice cold 7% perchloric acid [31,37,38,39]. The samples were vortexed for 30 s and sonicated on ice for 5 min. The vortex-sonication cycle was repeated three times. The samples were centrifuged at 14,500 rpm for 3 min at room temperature. The supernatant was taken out and neutralized to pH 7 with 3 M NaOH and 1 M phosphate buffer pH 9. The neutralized samples were centrifuged again at 14,500 rpm for 3 min. Clear supernatants were injected into HPLC to separate NAD^+^ from other cellular components. NAD^+^ peaks were collected according to the retention time of the authentic standard. Collections were lyophilized to dryness and subjected to MALDI-TOF analysis. Ratios of intensities for *m*/*z* = 664, 665, and 666 peaks, corresponding to unlabeled-, ^15^N-, and ^18^O-NAD^+^ isotopomers, were used to calculate NAD^+^ concentration in the sample. Corrections were applied for isotopic abundance.

### 2.5. Inhibition Study in Parasite Lysates

Synchronized parasites were incubated for 36 h under standard conditions (media were changed at the 24 h time point). Then they were treated with either vehicle (DMSO) or 50 μM 5-Me-nicotinaldehyde for 12 h. After a total of 48 h incubation (36 h drug free and 12 h drug-administrated), parasites were harvested using percoll/sorbitol gradient and washed twice with complete media. The pellets were then washed with 1 mL of PBS and were resuspended in 1 mL of PBS for cell counting using hemocytometer. After counting, the cells were centrifuged at 4000 rpm for 3 min at room temperature. To the pellets were added 60 μL of lysis buffer (50 mM Tris-HCl, pH 7.5, 5 mM EDTA and 1% Triton X-100), the pellets were gently suspended in the buffer. The samples were centrifuged at 4000 rpm for 3 min at room temperature. The supernatants were taken out and treated with 260,000 cpm of carbonyl ^14^C-nicotinamide for 30 min at 37 °C. Nicotinamide and nicotinic acid were separated and collected by HPLC, and radioactivity was determined by scintillation counting.

### 2.6. Luciferase Assay

The NF54-*luc* strain (a chloroquine sensitive line) expresses the *Renilla* luciferase gene under the control of the constitutively active *hrp3* promoter [40]. It was a generous gift from Dr. Kirk Deitsch (Weill Cornell Medical College). For viability assays, parasites were plated at 1–10% parasitemia in 96-well plates in standard media in the presence of the indicated concentrations of inhibitor. The total volume of culture was 200 μL and DMSO did not exceed 1% of the total culture volume. Media plus compounds were replenished every 12 h. After 48 h incubation, media were withdrawn, and RBCs were lysed with 100 μL of Bright-Glo Lysis Buffer (Promega, Madison, WI, USA). Lysates were then transferred to 96-well luminometer plates. To the lysates were added 100 μL of Bright-Glo *Renilla* Luciferase Reagent (Promega, Madison, WI, USA). Luminescence was measured immediately in a plate luminometer (Molecular Devices, San Jose, CA, USA). Each individual data point is represented in triplicate allowing determination of standard deviations. A broad range of compound concentrations are used to allow the determination of IC_50_ value.

### 2.7. Preparation of Giemsa Stained Smears

Ready-to-use Giemsa stain solution (Sigma-Aldrich, St. Louis, MO, USA) was diluted 1:20 in phosphate buffer (pH~7). The blood smear was air-dried and fixed in methanol for 30 s. The slide was then placed in a staining jar containing the freshly made Giemsa working solution. The slide was stained for 15 min before it was rinsed in deionized water. The slide was then air-dried and observed with immersion oil.

## 3. Results

### 3.1. Essential Catalytic Residues Are Conserved in P. falciparum Nicotinamidase (PfNic)

**Figure 3:**
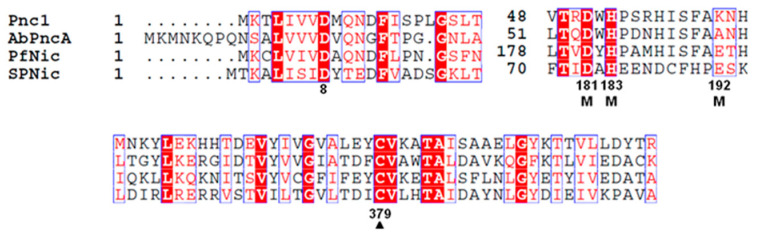
ClustalW alignment of the catalytic residues of nicotinamidases from *S. cerevisiae* (Pnc1), *A. baumanii* (AbPncA), *P. falciparum* (PfNic), and *S. pneumoniae* (SpNic). Identical residues are highlighted. The proposed metal binding residues are shown with an M underneath.

Nicotinamidase has been found in numerous organisms. With the advent of genome sequencing, it is now possible to compare phylogenetic similarities of nicotinamidases derived from very different organisms (Figure 3). Common features of the nicotinamidases include a catalytic triad composed of a universally conserved Asp, found near the N-terminus of most nicotinamidases, a catalytic Cys, and a universally conserved Lys residue [34,41,42,43,44,45]. In addition, a feature of these active sites is a cis-peptide that creates the oxyanion hole that stabilizes tetrahedral intermediates generated during catalysis. Furthermore, a conserved metal ion binding site is found on all known nicotinamidases. Highly conserved His residues compose part of the binding site on all known nicotinamidase structures. For *S. cerevisceae* [44], *S. pnemoniae* [41], and *A. baumanii* [42], the metal bound is a transition metal ion, most likely Zn^2+^. This ion has been proposed to be a Mn^2+^ or Mn^2+^/Fe^2+^ for the M. tuberculosis nicotinamidase [43,46]. In fact, Mn^2+^ appears to be able to substitute for Zn^2+^ on the *S. pneumoniae* enzyme [34].

### 3.2. Expression and Characterization of PfNic

In PlasmoDB, a gene producing a transcript called PFC0910w was cloned into a cloning vector. The transcript encodes a protein of predicted length of 429 amino acids (AAs) and molecular weight of 50.1 kDa. The typical nicotinamidase domain from most organisms is much shorter, comprising approximately 200 AAs, indicating that the *P. falciparum* enzyme might have an unusual structure. Subcloning into pet28a, which confers a histidine tag at the N-terminus, enabled protein expression in Rosetta (DE3) *E. coli* cells [34]. The isolated protein was purified to homogeneity by Ni-resin chromatography (Figure S1). Activity assay of this protein confirmed it to be a potent nicotinamidase, with steady state kinetic parameters of K_m_ = 3.0 ± 0.9 μM and k_cat_ = 0.35 ± 0.028 s^−1^ at pH 7, consistent with the previous report [34].

### 3.3. Identification of PfNic Inhibitors

In 1987, nicotinaldehyde was reported to be a weak inhibitor of the *S. cerevisceae* nicotinamidase, with a K_i_ value reported to be 68 μM [47]. This finding was later re-investigated to determine that nicotinaldehydes are general and very potent inhibitors of nicotinamidases [34]. These compounds form enzyme-stabilized hemithioacetals as proven on the *S. pneumoniae* nicotinamidase by X-ray crystallography [41]. The K_i_ values of nicotinaldehyde were found to be in the range 11–1400 nM for *S. pneumoniae*, *S. cerevisceae*, *P. falciparum*, B. burgdorferi, and two *C. elegans* enzymes [34]. The aldehyde inhibitions were fully reversible and competitive with nicotinamide substrate [34]. A series of 3-pyridinecarboxaldehydes were synthesized and evaluated for their ability to inhibit nicotinamidases. These structure–activity relationship (SAR) studies have significantly improved our knowledge on the design and development of nicotinamidase inhibitors [34]. The small molecule nicotinamidase inhibitors provide new tools for the analysis of NAD^+^ biosynthetic pathways in *P. falciparum*. A potent lead compound, 5-methyl-nicotinaldehyde (5-Me-nicotinaldehyde), was chosen for further in vitro studies.

### 3.4. 5-Me-Nicotinaldehyde Inhibits PfNic Activity in P. falciparum Lysates

**Figure 4:**
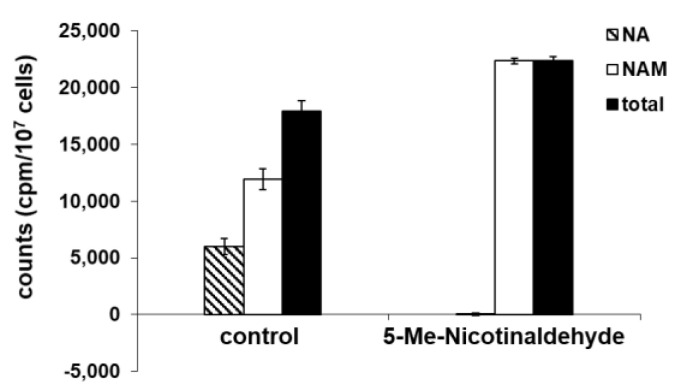
Inhibition of nicotinamidase in *P. falciparum* parasite lysate. Synchronized parasites were treated with or without 50 μM 5-Me-nicotinaldehyde for 12 h; harvested parasites were lysed and treated with 260,000 cpm of carbonyl ^14^C-nicotinamide for 30 min. NAM and NA were separated and collected by HPLC; radioactivity was determined by scintillation counting. For the drug treated parasites, there is no significant conversion of NAM to NA, indicating 5-Me-nicotinaldehyhde was able to inhibit *P. falciparum* nicotinamidase in the lysate. Error bars represent S.D. of at least three biological replicates.

The function of PfNic is to proteolytically convert NAM to NA. By using carbonyl ^14^C-labeled NAM, this enzymatic hydrolysis can be monitored by HPLC and scintillation counting as described in Section 2. Using this assay, we found that *P. falciparum* lysates efficiently processed NAM (Figure 4). Furthermore, this activity can be blocked by pre-incubation of parasites with 50 μM 5-Me-nicotinaldehyde for 12 h (Figure 4). The mechanism of nicotinaldehyde inhibition is likely conserved for nicotinamidases and was established to occur via thiohemiacetal formation [34,41]. Inhibition by 5-Me-nicotinaldehyde suggests that NAM processing observed in parasite lysates is the result of active PfNic.

### 3.5. Nicotinamide Is the Preferred NAD^+^ Precursor in P. falciparum

The elucidation of NAD^+^ biosynthesis in cultured *P. falciparum* parasites is crucial to improve understanding of the feasibility of using inhibition of nicotinamidase as a strategy to prevent NAD^+^ biosynthesis and to control or prevent *P. falciparum* growth. Synchronized *P. falciparum* parasites growing in red blood cells (RBCs) in 20 mL culture volumes with 100 μM exogenously added NAM were incubated for 48 h (the blood stage *P. falciparum* typically demonstrates a 48 h life cycle) [48]. Percoll/sorbitol gradient separation allowed the recovery of 95% schizonts. NAD^+^ contents in these cells were measured by adding ^18^O-NAD^+^ and processed for MS analysis as reported previously [31,49,50]. The intensity of the peak for ^18^O-NAD^+^ (*m*/*z* = 666) was ratioed against peak of ^16^O-NAD^+^ (*m*/*z* = 664) in MALDI-MS to determine the NAD^+^ concentration in these cells to be 53 pmol/10^6^ cells. The NAD^+^ content was also determined in recovered uninfected RBCs to be 10.7 pmol/10^6^ cells. The 5 fold increase of NAD^+^ in the infected RBCs is in agreement with the report that *P. falciparum* causes an approximately 10 fold increase in NAD^+^ synthesis is infected RBCs [33].

**Figure 5:**
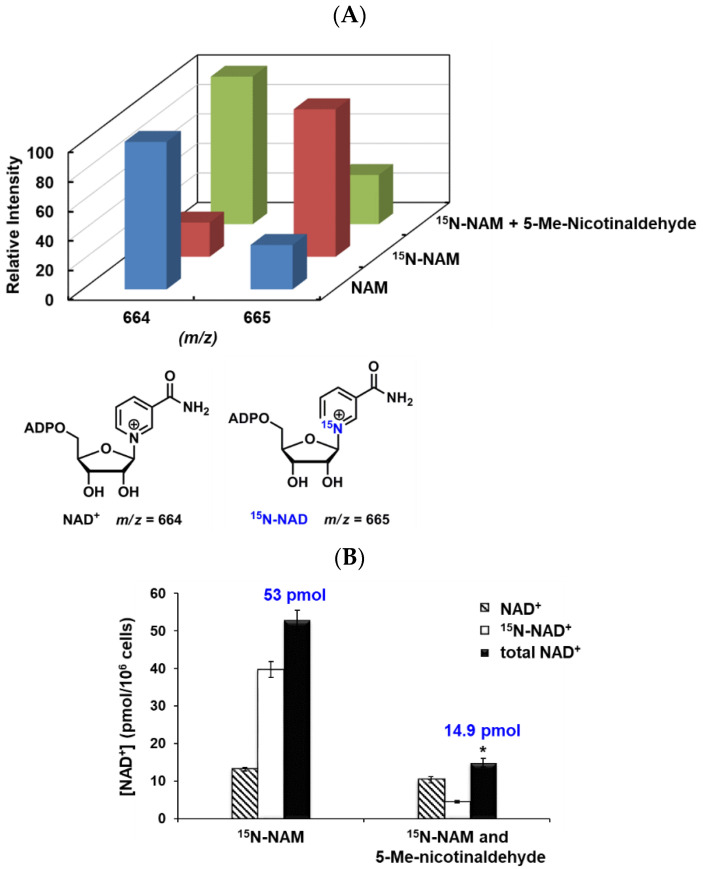
NAM is the preferred precursor for *P. falciparum* NAD^+^ biosynthesis. (**A**) Relative intensity of NAD^+^ isotopomers from parasite culture treated with 100 μM NAM, 100 μM ^15^N-NAM, or 100 μM ^15^N-NAM and 50 μM 5-Me-nicotinaldehyde for 48 h. NAD^+^ levels were determined by MALDI-MS. The chemical structures of NAD^+^ and ^15^N-NAD^+^ are shown below. (**B**) The addition of 5-Me-nicotinaldehyde, a nanomolar inhibitor of *P. falciparum* nicotinamidase, decreased the conversion of ^15^N-NAM to ^15^N-NAD^+^ to only 10% of inhibitor free sample. Addition of the inhibitor also caused total NAD^+^ level to decrease from 53 pmol/10^6^ cells to 14.9 pmol/10^6^ cells (* *p* = 0.003). Error bars represent S.D. of at least three biological replicates.

To determine the percentage of NAD^+^ that is synthesized from NAM versus any other precursor, we used ^15^N labeled NAM as the source of isotopically labeled precursor. 1-^15^N-NAM (99%) and 1-^15^N-NA (99%) were synthesized as described before [51,52]*. P. falciparum* culture already contains 8.2 μM unlabeled NAM (in RPMI 1640) [53] and 10 μM unlabeled NA (an ingredient in Albumax II as determined by us, Figure S2). We supplemented the media with either 100 μM unlabeled NAM or 100 μM ^15^N-NAM (Figure 5A). The highest intensity peak in each case was normalized to be 100; the ratio of 664 peak to 665 peak reflects the relative amount of NAD*^+^* vs. *^15^*N-NAD*^+^*. When treated with regular NAM, the intensity of 665 peak is minimal which represents the natural abundance of this isotopomer. After 48 h, the ^15^N-NAM treated cells have nearly 80% ^15^N incorporation in NAD^+^, indicating ^15^N-NAM is the dominant precursor for NAD^+^ biosynthesis under these conditions. When parasites were treated with 20 μM ^15^N-NA and 20 μM unlabeled NAM, NAD^+^ levels remained at 50 pmol/10^6^ cells without significant incorporation of ^15^N was observed (Figure S3). These data support the hypothesis that NAM is the dominant source of *P. falciparum* NAD^+^ in culture.

### 3.6. Inhibition of NAD^+^ Biosynthesis by Nicotinamidase Inhibitors Can Be Reversed by Nicotinic Acid

To test if an inhibitor of *P. falciparum* nicotinamidase could decrease the conversion of NAM to NAD^+^, we incubated the parasites with 100 μM ^15^N-NAM with or without 50 μM 5-Me-nicotinaldehyde. As shown in Figure 5B, the presence of PfNic inhibitor caused 90% loss of ^15^N incorporation in NAD^+^. Although recovered cell counts after 48 h were similar for both groups, the inhibitor treated parasites experienced a statistically significant loss of NAD^+^ down to only 28% of controls (*p* = 0.003, Figure 5B). These suggested that *P. falciparum* is highly dependent upon nicotinamidase for NAD^+^ homeostasis and that interference with this enzyme can cause acute depletion of NAD^+^ level.

**Figure 6:**
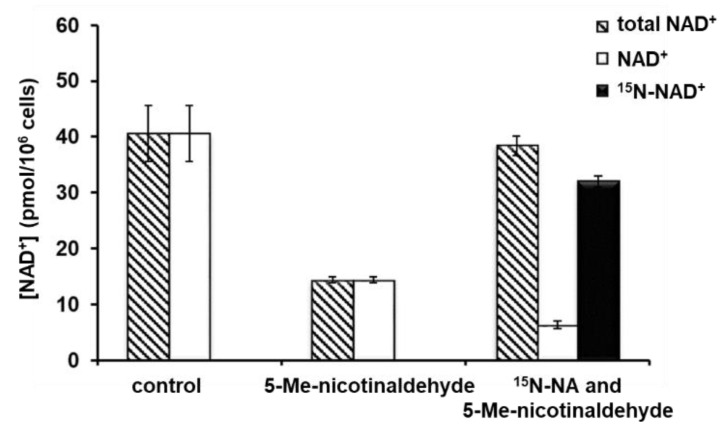
The disrupted NAD^+^ homeostasis through inhibition of nicotinamidase can be corrected with nicotinic acid. NAD^+^, ^15^N-NAD^+^, and total NAD^+^ levels in parasites treated with vehicle (DMSO), 50 μM 5-Me-nicotinaldehyde, or a combination of 50 μM 5-Me-nicotinaldehyde and 100 μM ^15^N-NA. The significant decrease of NAD^+^ concentration upon inhibitor administration was rescued by the addition of ^15^N-NA with 81% ^15^N incorporation in NAD^+^. Error bars represent S.D. of at least three biological replicates.

Furthermore, in order to rule out the possibility of off-target effect and to determine if a defect in NAD^+^ biosynthesis can be rescued by a downstream metabolite, parasites were treated with either vehicle (DMSO), or 50 μM 5-Me-nicotinaldehyde, or a combination of 50 μM 5-Me-nicotinaldehyde and 100 μM ^15^N-NA. Indeed, the significant decrease of NAD^+^ concentration upon inhibitor administration was rescued by the addition of a high dose of NA with 81% ^15^N incorporation in NAD^+^ (Figure 6). These experiments allowed us to assess the specific repressive effect of PfNic inhibitor on NAD^+^ level. The disrupted NAD^+^ homeostasis can be corrected with another NAD^+^ precursor, namely, NA.

### 3.7. Nicotinaldehydes Inhibits P. falciparum Replication

**Figure 7:**
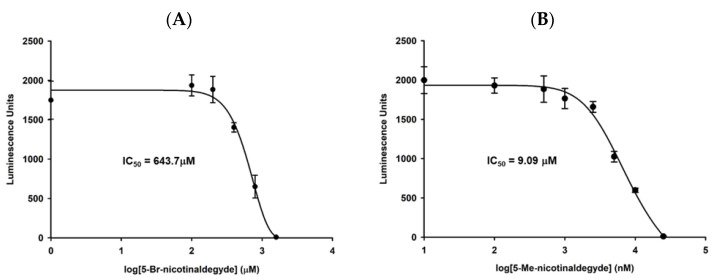
Viability assay for parasites treated with nicotinaldehyde derivatives. 3D7 Parasites (chloroquine-sensitive) were plated at 1–10% parasitemia in 96-well plates in standard media in the presence of the indicated concentrations of inhibitor. The total volume of culture was 200 μL, and DMSO did not exceed 1% of the total culture volume. Media plus compounds were replenished every 12 h. After 48 h incubation, media were withdrawn and RBCs were lysed with 100 μL of Bright-Glo Lysis Buffer (Promega, Madison, WI, USA). Lysates were then transferred to 96-well luminometer plates. To the lysates were added 100 µL of Bright-Glo *Renilla* Luciferase Reagent (Promega, Madison, WI, USA), luminescence was measured immediately in a plate luminometer (Molecular Devices). Each individual data point is represented in triplicate allowing determination of standard deviations. A broad range of compound concentrations are used to allow the determination of IC_50_ value to be 643.7 μM for 5-Br-nicotinaldehyde (**A**), and 9.09 μM for 5-Me-nicotinaldehyde (**B**), respectively. Error bars represent S.D. of two independent experiments in triplicates.

**Figure 8:**
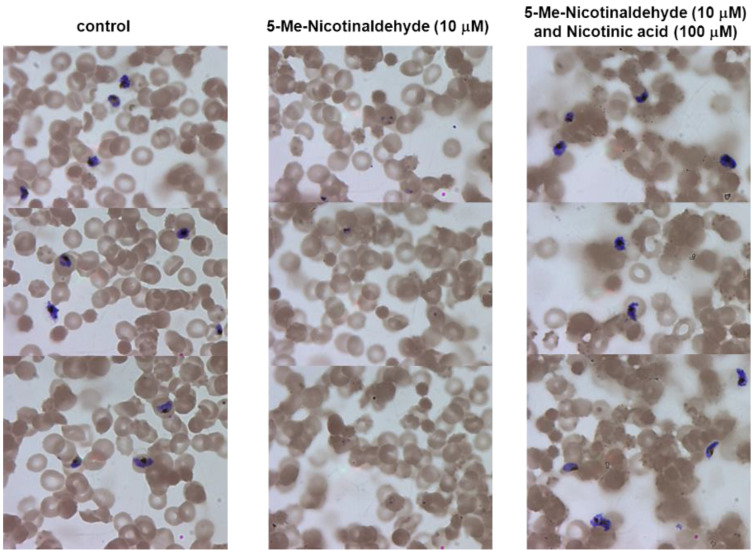
Inhibitory effect of 5-Me-nicotinaldehyde *in vitro*. Images of Giemsa-stained blood smears from synchronously grown parasites cultures treated with DMSO (control), 10 μM 5-Me-nicotinaldehyde or 10 μM 5-Me-nicotinaldehyde plus 100 μM NA. Smears were taken 48 h after the addition of the compound. Culture media contained only 0.1% Albumax II, this would reduce the nicotinic acid level in the media down to 2 μM. *Pf*Nic inhibitor caused growth arrest of parasites in the ring stage, this arrest can be reversed with the addition of NA. The treatments were repeated in three biological replicates.

5-Br-nicotinaldehyde was tested for its ability to inhibit chloroquine-sensitive *P. falciparum* strain 3D7 growth using both the *Renilla*-luciferase assay [54] to measure parasites viability, and microscopic examination of morphological changes. Although the compound did not significantly affect parasite’s growth at moderate concentrations (IC_50_ = 643.7 μM) (Figure 7A), it did exert inhibitory effect on NAD^+^ level (Figure S4). We reasoned that the presence of 10 μM NA in regular culture medium (0.5% Albumax II) could rescue the metabolic block induced by *Pf*Nic inhibitors. Reducing NA level down to 2 μM in culture media (0.1% Albumax II) did not affect normal growth in the 48 h time frame (Figure S5). With reduced Albumax II content, the incubation with 200 μM 5-Br-nicotinaldehyde completely blocked host cell escape and reinvasion by arresting parasites in the ring growth stage (Figure S6). The more potent compound, 5-Me-nicotinaldehyde with an IC_50_ value of 9.09 μM (Figure 7B), demonstrated a similar effect at 10 μM (Figure 8), and more importantly, this growth arrest can be reversed with the addition of 100 μM NA. These data confirm that depletion of NAD^+^ is detrimental to *P. falciparum*, and inhibition of *Pf*Nic is indeed effective to block parasite replication. It is important to note that the IC_50_ values of the inhibitors may be altered in response to different Albumax II contents (different NA concentrations).

## 4. Discussion and Conclusions

The idea that nicotinamidase could represent an attractive antimicrobial target in pathogens that infect humans is supported by genetic studies [55,56,57]. In organisms with NAD^+^ metabolism similar to *P. falciparum*, the loss of nicotinamidase achieved by genetic deletion caused loss of virulence, infectivity and proliferative capability. For example, *Brucella abortus* infects phagocytes and lives within them [56,58]. These are gram negative bacteria and cause chronic infections, in part because of their intracellular lifecycle, which enables them to evade host immunity. *B. abortus* lacks a de novo NAD^+^ biosynthetic pathway. It has nicotinamidase, nicotinate mononucleotide adenylyltransferase (NMNAT), nicotinamide phosphoribosyltransferase (NAMPT), and NAD^+^ synthetase genes, similar to what has been found for *P. falciparum.* Removal of the nicotinamidase gene (*PNCA*) from *B. abortus*, one of four *Brucella* species known to infect humans, causes a nearly complete failure in intracellular replication in culture unless media are supplemented with NA. [59]. Similarly, growth in macrophages is strongly attenuated in *PNCA*^−^ bacteria [59]. *PNCA^−^* bacteria cause an appropriate immune response suggesting *PNCA* deletion may represent a means for an attenuated live vaccine for *Brucella* [59]. These examples emphasize the likelihood that nicotinamidase inhibitors could represent viable compounds to limit pathogen growth and virulence in humans, providing data in support of the idea that targeted inhibition of nicotinamidase in organisms such as *P. falciparum,* which have similarly configured NAD^+^ biosynthetic pathways, could represent an effective yet unexplored therapeutic approach.

Up until recently, both the catalytic mechanism and the structural organization of the active sites of nicotinamidases were essentially unknown, and there were no known general inhibitors of these enzymes. This situation has changed with the development of a continuous assay for nicotinamidase activity, the identification of nicotinaldehydes as potent and general mechanism-based inhibitors of nicotinamidases, and the X-ray crystal structural studies of liganded nicotinamidases [34,41]. These recent findings have clarified important features of the active site design and crucial aspects of the catalytic mechanism.

The *P. falciparum* nicotinamidase was cloned and functionally expressed. Kinetic properties of this enzyme along with inhibitions by nicotinaldehydes have been reported [34]. In this study, we demonstrated that 5-Me-nicotinaldehyde inhibits *Pf*Nic activity in parasite lysates. To monitor the metabolites utilization by the parasites, we employed isotopically labeled NAD^+^ precursors and used NAD^+^ levels and isotope abundance as readouts. 5-Me-nicotinaldehyde causes dramatic reduction of the incorporation of NAM into NAD^+^ and significant depletion of NAD^+^ level in parasite cultured in human RBCs. This reduction can be restored by a high concentration of NA. These studies not only elucidate the NAM salvage pathway in *P. falciparum* but also support the hypothesis that NAM is the preferred precursor for NAD^+^ along this pathway.

An important assumption about the effectiveness of nicotinamidase inhibition as a potential antimalarial approach is the limited availability and bioactivity of NA in the body. Indeed, although nicotinaldehydes showed an inhibitory effect on NAD^+^ concentrations and dose-dependent inhibition of parasites growth, these effects were not sufficient to fully block parasite replication in our initial tests. Only when nicotinic acid level was reduced by 80% in the culture medium, in other words, the availability of alternative NAD^+^ precursor was minimized, growth arrests were observed upon inhibitor treatment. In human serum, the NA level is around 0.1~1 μM [60,61]. Dietary NA derives mostly from plant foods, is converted to NAD^+^ predominantly in the intestine and liver, and is released to the rest of the body from the liver in the form of NAM [62,63]. For erythrocytes that harbor *P. falciparum* parasites, measurements suggest such low concentrations of NA in these cells that they are virtually undetectable. The cumulative amount of NaMN and NaAD is only 6 μM, whereas NMN and NAD^+^ levels cumulatively represent 400 μM [64], suggesting that NAM is likely the most available source for the pyridine-3-carboxy moiety for *P. falciparum* in the host.

The nicotinamidases from the species *P. berghei* and *P. yeolii* exhibit 53% identity to *Pf*Nic (Figure S7). Thus, it could be possible to have new antimalarial drugs that are effective against major species of *Plasmodium* by targeting nicotinamidases.

Cells under stress due to treatment with antibiotics or chemotherapeutic drugs often require increased NAD^+^ levels for recovery. Thus, compounds that reduce NAD^+^ production often act synergistically with drugs that target other, unrelated metabolic pathways. This is particularly true for drugs that are known to release reactive radicals or cause oxidative damage inside the cell. Several antimalarial drugs, including chloroquine, mefloquine, and quinine, are thought to cause extensive oxidative damage by disrupting heme polymerization [65]. There is a high probability that reducing NAD^+^ levels in the cells will make the parasites hypersensitive to these compounds. Therefore, nicotinamidase inhibitors might act synergistically with these drugs in treating malaria.

The action of nicotinamidases on NAD^+^ levels inside parasites can have profound effects on gene expression. The *P. falciparum* genome contains two *sir2* genes that encode partially redundant versions of the protein SIR2 [66]. Knockouts of either of these genes result in down-regulation of the overall level of SIR2 activity in the cell and lead to significant de-silencing of large portions of the *var* gene family [67,68]. Each *var* gene encodes a different variant of the surface protein erythrocyte membrane protein 1 (*Pf*EMP1), the primary virulence determinant of *P. falciparum*. The parasite genome contains 60 *var* gene copies, with all but a single gene maintained in a silent state, at least in part through the action of SIR2. It is important for each parasite to only express one *var* gene at a time, and to switch expression over the length of an infection to enable it to avoid the host’s antibody response against previously expressed *var* gene products [69]. The parasite’s ability to maintain a chronic infection is dependent on this coordinated gene expression pattern, and disruption of *var* gene silencing would expose the entire antigen repertoire to the host prematurely, thus effectively “vaccinating” the infected individual against all variants of the parasite. Disruption of silencing of an analogous gene family in the intestinal parasite *Giardia lamblia* did indeed result in the effective vaccination of infected mice [70], lending support for the potential efficacy of this strategy. Since treatment with nicotinamidase inhibitors results in drastic reduction in NAD^+^ levels, it is likely that SIR2 activity will be reduced correspondingly and that *var* gene silencing might be disrupted. All of these await further investigation.

## Data Availability

Not applicable.

## Supplementary Materials

The following supporting information can be downloaded at: https://www.mdpi.com/article/10.3390/biom12081109/s1, Figure S1: SDS-PAGE of purified *Pf*Nic; Figure S2: *P. falciparum* culture medium contains NA; Figure S3: NA is not the preferred NAD biosynthetic precursor for *P. falciparum*; Figure S4: 5-Br-nicotinaldehyde reduces *P. falciparum* NAD^+^ levels; Figure S5: Reduced Albumax II concentration does not affect parasite growth; Figure S6: Inhibitory effect of 5-Br-nicotinaldehyde *in vitro*; Figure S7: Sequence alignment of nicotinamidases from *P. Berghei*, *P. Yoelli*, and *P. falciparum*.
