# Supplementary material for: Plasmodium falciparum Nicotinamidase as A Novel Antimalarial Target
Source: Biomolecules. 2022 Aug 12;12(8):1109. doi: 10.3390/biom12081109 (PMC9405955; doi:10.3390/biom12081109)
Supplement: Supplementary file 1 [file biomolecules-12-01109-s001.zip › biomolecules-1825603-supplementary.pdf]

Supporting Information

Plasmodium falciparum Nicotinamidase as A Novel Antimalarial Target

Dickson Donu,[1] Chiranjeev Sharma,[1] Yana Cen[1,2,*]

[1]*Department of Medicinal Chemistry, Virginia Commonwealth University, Richmond, VA 23219*

[2]*Institute for Structural Biology, Drug Discovery and Development, Virginia Commonwealth University, Richmond, VA 23219*

[*]Correspondence: ceny2@vcu.edu, phone: 804-828-7405

Figure S1. SDS-PAGE of purified *Pf*Nic.

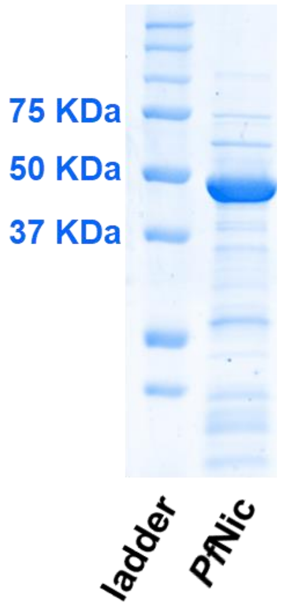

The gene PFC0910W was cloned into the protein expression vector pet28a. The pet28a-*Pf*Nic was transfected into Rosetta (DE3) *E. coli* cells. The protein was expressed and purified as described previously.[1] Protein concentration was determined using Bradford assay. The protein was >95% pure as determined with SDA-PAGE.

Figure S2. *P. falciparum* culture medium contains NA.

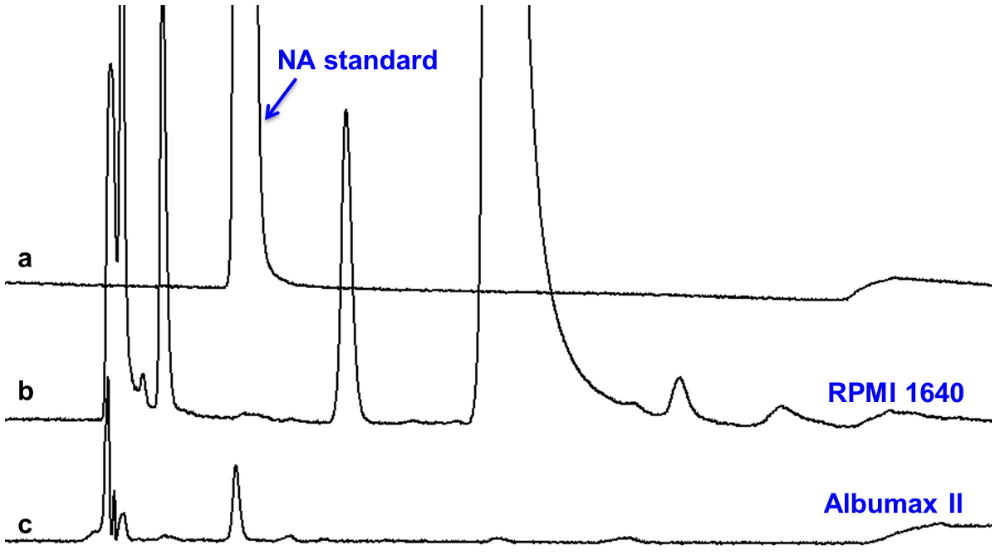

HPLC chromatograms showing that *P. falciparum* culture medium contains NA. Trace a is the chemical standard of NA. Traces b and c are the contents in RPMI 1640 and Albumax II. The concentration of NA in Albumax II was determined to be 10 μM. Briefly, Albumax II was lyophilized to dryness, and reconstituted in distilled water. To the sample was added 1 nmol of ^{15}N-NA along with 70 μL of ice cold 7% perchloric acid. The sample was vortexed for 30 s and sonicated on ice for 5 min. The vortex-sonication cycle was repeated three times. The sample was centrifuged at 14,500 rpm for 3 min at room temperature. The supernatant was taken out and neutralized to pH 7 with 3 M NaOH and 1 M phosphate buffer pH 9. The neutralized sample was centrifuged again at 14,500 rpm for 3 min. Clear supernatants were injected into HPLC to separate NA from other components. NA peaks were collected according to the retention time of the authentic standard. Collections were lyophilized to dryness and subjected to MALDI-TOF analysis. Ratios of intensities for m/z = 123 and 124 peaks, corresponding to unlabeled- and ^{15}N-NA isotopomers, were used to calculate NA concentration in the sample. Corrections were applied for isotopic abundance.

Figure S3. NA is not the preferred NAD biosynthetic precursor for *P. falciparum*.

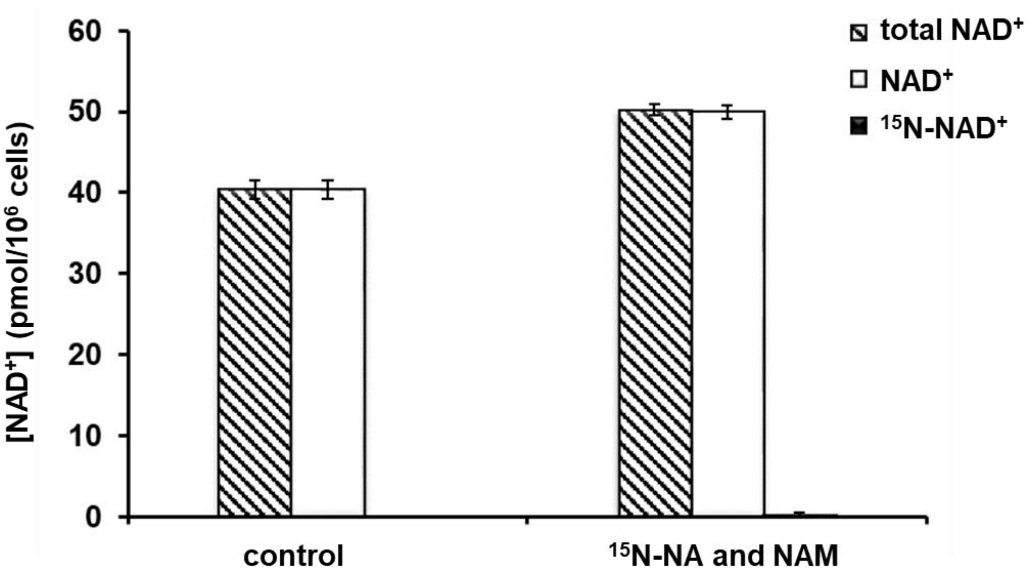

NAD$^+$, ^{15}N-NAD$^+$ and total NAD$^+$ levels in parasites treated with vehicle, or a combination of 20 μM ^{15}N-NA and 20 μM unlabeled NAM. The total NAD$^+$ level increased upon precursor treatment without significant incorporation of ^{15}N label. Error bars represent S.D. of at least three biological replicates.

Figure S4. 5-Br-nicotinaldehyde reduces *P. falciparum* NAD^+ levels.

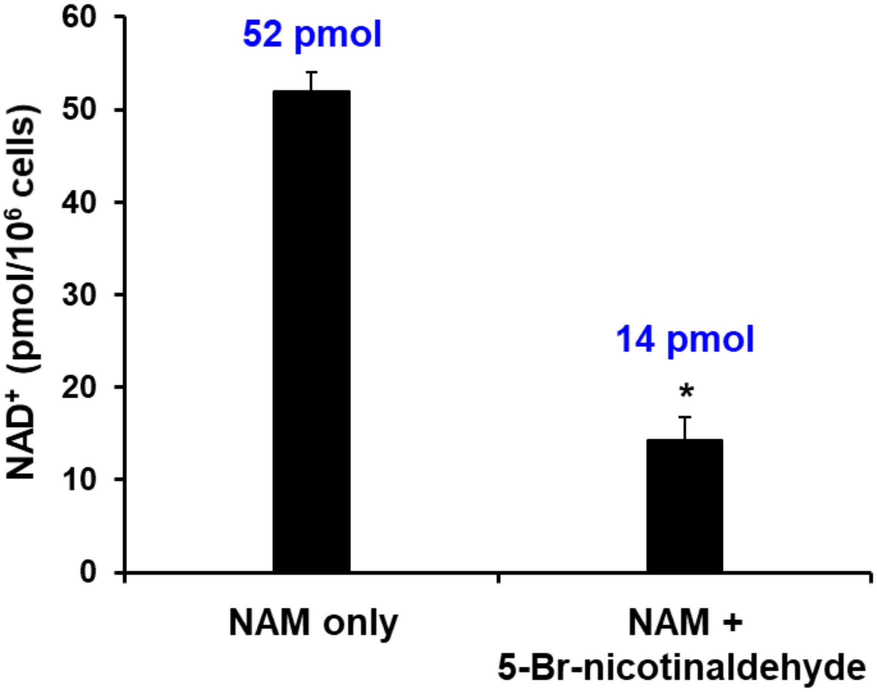

Total NAD^+ levels in parasites treated with 100 μM nicotinamide, or a combination of 100 μM nicotinamide and 200 μM 5-Br-nicotinaldehyde for 48 h, as determined by MALDI-MS (*$p < 0.003$). Error bars represent S.D. of at least three biological replicates.

Figure S5. Reduced Albumax II concentration does not affect parasite growth.

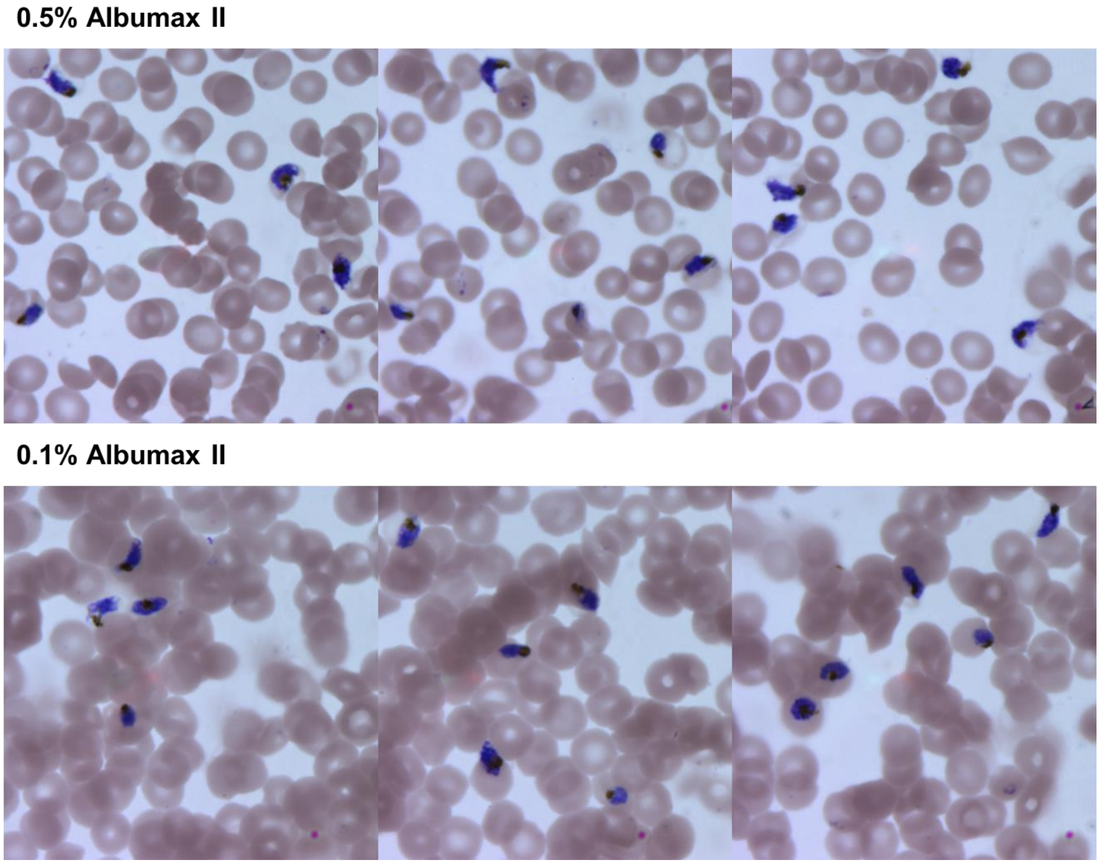

Images of Giemsa-stained blood smears from synchronously grown parasites cultures with 0.5% Albumax II (top panel), or with 0.1% Albumax II (bottom panel). Smears were taken 48 h after the start of the experiment. The reduced of Albumax II concentration caused negligible changes to parasite growth.

Figure S6. Inhibitory effect of 5-Br-nicotinaldehyde *in vitro*.

control | **5-Br-Nicotinaldehyde (200 μM)** | **5-Br-Nicotinaldehyde (200 μM) and Nicotinic acid (100 μM)**

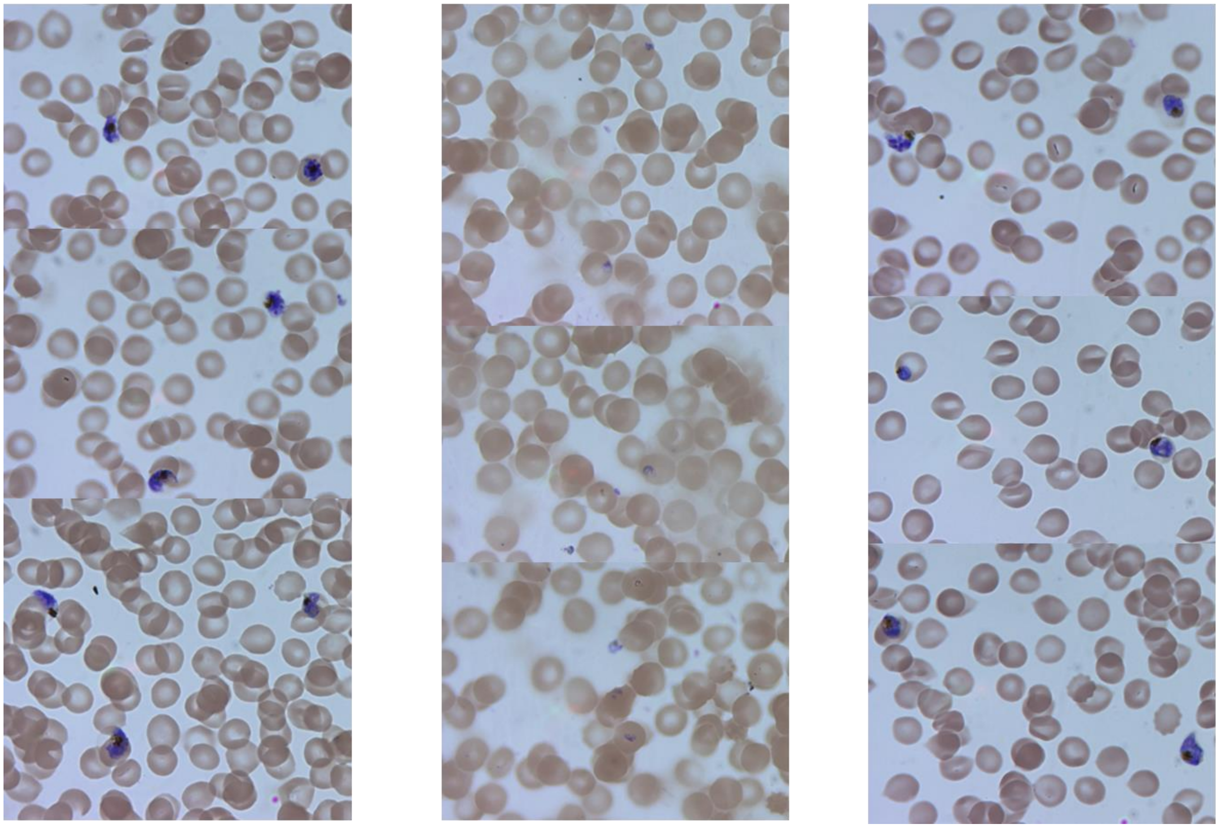

Images of Giemsa-stained blood smears from synchronously grown parasites cultures (incubated in media with 0.1% Albumax II) treated with DMSO (control), 200 μM 5-Br-nicotinaldehyde or 200 μM 5-Br-nicotinaldehyde plus 100 μM NA. Smears were taken 48 h after the addition of the compound.

Figure S7. Sequence alignment of nicotinamidases from *P. Berghei*, *P. Yoelli*, and *P. falciparum*.

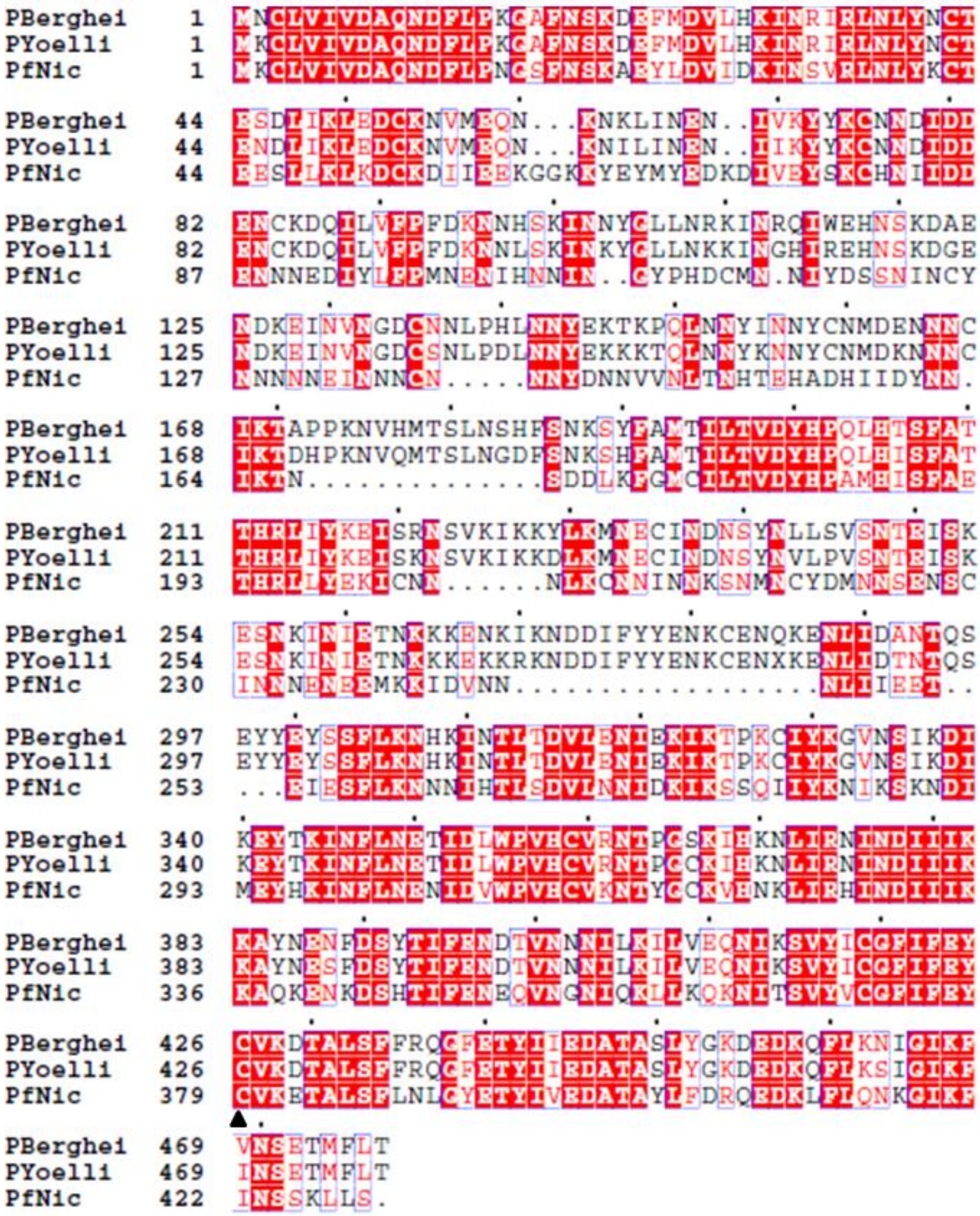

ClustalW alignment of nicotinamidases from *P. berghei*, *P. yoelli*, and *P. falciparum* (*Pf*Nic). Identical residues are highlighted.

REFERENCES

1. French, J. B.; Cen, Y.; Vrablik, T. L.; Xu, P.; Allen, E.; Hanna-Rose, W.; Sauve, A. A., Characterization of nicotinamidases: steady state kinetic parameters, classwide inhibition by nicotinaldehydes, and catalytic mechanism. *Biochemistry* **2010,** *49* (49), 10421-39.
